# Molecular and clinical analyses of *Helicobacter pylori* colonization in inflamed dental pulp

**DOI:** 10.1186/s12903-018-0526-2

**Published:** 2018-04-16

**Authors:** Ryota Nomura, Yuko Ogaya, Saaya Matayoshi, Yumiko Morita, Kazuhiko Nakano

**Affiliations:** Department of Pediatric Dentistry, Division of Oral Infection and Disease Control, Osaka University Graduate School of Dentistry, 1-8 Yamada-oka, Suita, Osaka, 565-0871 Japan

**Keywords:** *Helicobacter pylori*, Inflamed pulp, Nested PCR method, Human dental fibroblast cells

## Abstract

**Background:**

Recently, dental pulp has been considered a possible source of infection of *Helicobacter pylori* (*H. pylori*) in children. We previously developed a novel PCR system for *H. pylori* detection with high specificity and sensitivity using primer sets constructed based on the complete genome information for 48 *H. pylori* strains. This PCR system showed high sensitivity with a detection limit of 1–10 cells when serial dilutions of *H. pylori* genomic DNA were used as templates. However, the detection limit was lower (10^2^–10^3^ cells) when *H. pylori* bacterial DNA was detected from inflamed pulp specimens. Thus, we further refined the system using a nested PCR method, which was much more sensitive than the previous single PCR method. In addition, we examined the distribution and virulence of *H. pylori* in inflamed pulp tissue.

**Methods:**

Nested PCR system was constructed using primer sets designed from the complete genome information of 48 *H. pylori* strains. The detection limit of the nested PCR system was 1–10 cells using both *H. pylori* genomic DNA and bacterial DNA isolated from inflamed pulp specimens. Next, distribution of *H. pylori* was examined using 131 inflamed pulp specimens with the nested PCR system. In addition, association between the detection of *H. pylori* and clinical information regarding endodontic-infected teeth were investigated. Furthermore, adhesion property of *H. pylori* strains to human dental fibroblast cells was examined.

**Results:**

*H. pylori* was present in 38.9% of inflamed pulp specimens using the nested PCR system. *H. pylori* was shown to be predominantly detected in primary teeth rather than permanent teeth. In addition, samplings of the inflamed pulp were performed twice from the same teeth at 1- or 2-week intervals, which revealed that *H. pylori* was detected in most specimens in both samplings. Furthermore, *H. pylori* strains showed adhesion property to human dental fibroblast cells.

**Conclusion:**

Our results suggest that *H. pylori* colonizes inflamed pulp in approximately 40% of all cases through adhesion to human dental fibroblast cells.

## Background

*Helicobacter pylori* is a Gram-negative microaerophilic bacterium found in the stomach that is responsible for gastric diseases [1]. Though details regarding its transmission and infection source remain unclear, the most likely route of infection is through the oral cavity [2]. To detect *H. pylori* in oral cavity specimens, molecular biological technique has been applied [3]. However, it is not easy to estimate the actual infection rate as *H. pylori* detected in the oral cavity has been reported to be 0–100% [4].

To overcome the difficulty of the detection of *H. pylori* in the oral specimens, we previously designed novel primer sets based on the information of the complete genome for 48 *H. pylori* strains in the database [5]. We searched genes including 16S rRNA, *vacA*, *cagA*, *glmM* (*ureC*), and *ureA* because these genes were used for *H. pylori* detection in published PCR methods with high frequency [6–10]. Among these genes, six sequences of at least 20 consecutive nucleotides conserved among all strains were found only in *ureA*, which were selected as primer sets for the detection. These primer sets produced amplicons for genomic DNA across *H. pylori* strains, but did not for *Helicobacter pullorum* and *Helicobacter felis* strains, the closest related species to *H. pylori*.

Most studies regarding *H. pylori* detection from oral specimens have focused on dental plaque or saliva specimens [11, 12]. A recent study showed that *H. pylori* was isolated from endodontic-infected root canals of primary teeth [13]. In addition, we detected *H. pylori* bacterial DNA in inflamed pulp specimens using our novel PCR method [5]. In the present study, we refined our system using a nested PCR method, which is much more sensitive than the previous single PCR method. The nested PCR method was then applied to investigate the actual *H. pylori* distribution in inflamed pulp specimens as well as correlations between the detection of *H. pylori* and clinical information regarding endodontic-infected teeth. Furthermore, the adhesion property of *H. pylori* strains to human dental fibroblast cells, which is considered an important virulence factor of *H. pylori* detected in inflamed pulp, was also investigated.

## Method

### Bacterial strains and growth condition

*H. pylori* reference strain 26,695 (ATCC 700392), J99 (ATCC 700824), ATCC 51932, and *H. pullorum* ATCC 51802 were purchased from Summit Pharmaceuticals International Corporation (Tokyo, Japan). *H. felis* ATCC 49179 was kindly provided by Professor Masakazu Kita (Department of Microbiology, Kyoto Prefectural University of Medicine, Kyoto, Japan). All strains were cultured by the method described previously using blood agar plates (Becton Dickinson, Franklin Lakes, NJ, USA) at 37 °C for 3 days [5]. Then, single colonies were selected, inoculated in 5 ml tryptic soy broth (Difco Laboratories, Detroit, MI, USA), and incubated at 37 °C for 3-5 days under microaerophilic conditions.

### PCR system for *H. pylori* detection

**Fig. 1:**
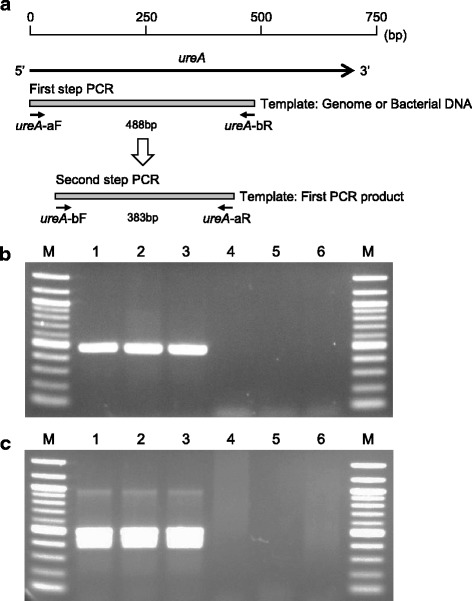
Nested PCR method for *H. pylori* detection. **a** A schematic diagram of positions of the designed primers in this study. **b** Representative images showing the single PCR assay for *H. pylori* detection using *ureA*-aF and *ureA*-bR primer sets. **c** Representative images showing the nested PCR assay for *H. pylori* detection using *ureA*-aF and *ureA*-bR primer sets, followed by *ureA*-bF and *ureA*-aR primer sets. Lanes: 1, *H. pylori* 26,695; 2, J99; 3, ATCC 51932; 4, *H. pullorum* ATCC 51802; 5, *H. felis* ATCC 49179; 6, sterile water. M, molecular size marker (100 bp DNA ladder)

*H. pylori* genomic DNA was extracted using a previously reported method for Gram-negative periodontitis-related bacteria [14]. We previously identified six sequences of at least 20 consecutive nucleotides in the *ureA* gene of 48 *H. pylori* strains registered in the GenBank database [5]. Based on these sequences, five primer sets (*ureA*-aF/aR, *ureA*-aF/bR, *ureA*-bF/aR, *ureA*-bF/bR, *ureA*-cF/cR) were constructed for PCR methods to detect *H. pylori* [5]. Among these primer sets, two primer sets for the first and second steps of the nested PCR method were selected as follows. First step PCR for *H. pylori* detection was performed using primers *ureA*-aF (5′-ATG AAA CTC ACC CCA AAA GA-3′) and *ureA*-bR (5′- CCG AAA GTT TTT TCT CTG TCA AAG TCT A-3′); second step PCR was performed using primers *ureA*-bF (5′-AAA CGC AAA GAA AAA GGC ATT AA-3′) and *ureA*-aR (5′-TTC ACT TCA AAG AAA TGG AAG TGT GA-3′) (Fig. 1a). First step PCR was performed by amplifying 2 μl template genomic DNA extracted from cultured *H. pylori* strains or bacterial DNA extracted from dental pulp specimens in reactions of 20 μl total volume. Second step PCR was performed by amplifying 1 μl of the first PCR product used as a template in reactions of 20 μl total volume. Both PCR cycles of first and second steps of the nested PCR methods were performed as the single PCR method, described previously using TaKaRa Ex *Taq* polymerase (Takara Bio. Inc., Otsu, Japan) [5].

### Specificity and sensitivity of the first and second step PCR systems

The specificity of primer sets for *H. pylori* detection was assessed using genomic DNA extracted from *H. pylori*, *H. pullorum*, and *H. felis*. The sensitivity of the PCR assays was determined using titrated cultures of *H. pylori* strain J99. The detection limits of the first step and second step PCR assays were determined using 10-fold serial dilutions of genomic DNA extracted from known numbers of bacterial cells. In addition, five inflamed pulp specimens were taken during root canal treatments. The total numbers of oral bacteria in these specimens were confirmed by plating serially diluted samples onto blood agar plates, which were anaerobically cultured at 37 °C for 4 days; specimens contained approximately 9 × 10^5^–4 × 10^6^ c.f.u. (mean 2.6 × 10^6^ c.f.u.). Among these samples, two *H. pylori*-negative specimens were selected, which were confirmed using the nested PCR method described above. Two dental pulp specimens without bacterial infection were also collected during extraction of completely impacted wisdom teeth. Serial dilutions of known bacterial number of *H. pylori* were added to the inflamed and non-inflamed pulp specimens, followed by DNA extraction. Then, the detection limits of the first and second step PCR assays were determined using bacterial DNA as template.

### Dental pulp specimens

This study was conducted in full adherence to the Declaration of Helsinki. The study protocol was approved by the Ethics Committee of Osaka University Graduate School of Dentistry (approval no. H23-E1-5). Prior to specimen collection, the subjects were informed of the study contents and written informed consent was obtained from all participants. When the participant was aged under 16, parental written informed consent was also obtained.

Inflamed pulp specimens were obtained from 131 subjects (age range: 1-19 years) who were treated at Osaka University Dental Hospital from February 2013 to February 2016. In our clinic, dental treatments including root canal treatments are performed children aged over 3 or 4 years. However, we performed the root canal treatments to the subjects with lower ages under restraint after the consent of the parents when the subjects have acute symptoms such as spontaneous pain or gingival abscess formation. The specimens were taken from patients who received root canal treatment under local anesthesia because of severe childhood caries (*n* = 114) or trauma (*n* = 17). The 101 and 30 specimens were obtained from primary and permanent teeth, respectively. Among 131 specimens, 36 were used in our previous study [5]. Inflamed pulp specimens taken from different teeth in the same patient were included among the 131 specimens and given a different specimen number.

Among 131 specimens, 20 specimens were taken additional sampling from the same teeth. All 20 specimens were collected from infected root canals with apical abscess formation. First samplings were performed in initial root canal treatments. When a gingival abscess formed, root canals were generally left unsealed and antibiotics were not applied during initial root canal treatments. Thus, second samplings were performed when the subjects visited the hospital for the second root canal treatment (*n* = 20). The detection frequencies of *H. pylori* between first and second samplings were compared.

The sampling method for dental pulp specimens was described previously [5]. The samples were stored on ice and immediately transported to our laboratory for testing. DNA extraction and nested PCR were performed as described above. To confirm that the amplified fragments in second step PCR were targeted species, ten positive bands were randomly selected and sequenced as described previously [15].

### Adhesion to human dental pulp fibroblast cells (HDPFs)

The adhesion property of *H. pylori* strains to HDPFs was examined using a method described previously [16], with some modifications. Approximately 1 × 10^5^ HDPFs were seeded in the tissue culture plates (Costar®, Corning Inc., Corning, NY, USA). The wells were washed with PBS and antibiotic-free medium was added, followed by the infection of 1 × 10^6^ c.f.u. *H. pylori* in antibiotic-free medium. The medium was removed after 1.5 h of anaerobic incubation and infected cells were washed with PBS, and then added the sterile distilled water for disruption of the cells. Next, dilutions of cell lysates were plated onto blood agar plates and incubated at 37 °C for 3 days under microaerophilic conditions. The adhesion rates were calculated by the ratio of resuspended to infected cells. Data are shown as the mean ± standard deviation of triplicate experiments.

### Statistical analyses

Statistical analyses were performed using the computational software package GraphPad Prism 6 (GraphPad Software Inc., La Jolla, CA, USA). Intergroup differences in each analysis were analyzed using Bonferroni’s method after analysis of variance (ANOVA). A *P* value of less than 0.05 was considered to be statistically significant.

## Results

### Specificity and sensitivity for the first and second step PCR systems

**Fig. 2:**
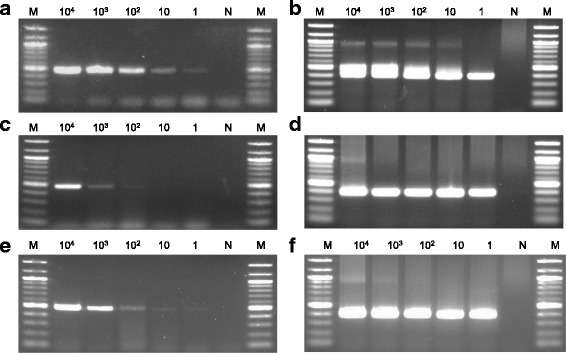
Sensitivity of the single and nested PCR assays for *H. pylori* detection. Representative images showing the sensitivities of (**a**) single and (**b**) second step PCR assays for the detection of *H. pylori* J99 genomic DNA. Representative images showing the sensitivities of (**c**) single and (**d**) second step PCR assays for the detection of *H. pylori* bacterial DNA from inflamed pulp. Representative images showing the sensitivities of (**e**) single and (**f**) second step PCR assays for the detection of *H. pylori* bacterial DNA added to non-infected dental pulp. The *ureA*-aF and *ureA*-bR primer sets were used for the single PCR assay and *ureA*-bF and *ureA*-aR primer sets were used for the second step PCR assay. M, molecular size marker (100 bp DNA ladder)

The first and second step PCR primer sets produced amplicons consistent in size for genomic DNA of all *H. pylori* strains, but were not seen in *H. pullorum* and *H. felis* strains (Fig. 1b, c). The first and second step PCR primer sets showed a level of sensitivity at approximately 1–10 c.f.u. per reaction when *H. pylori* genomic DNA was used as template (Fig. 2a, b). However, the sensitivity of the first step PCR primer sets was decreased (10^2^–10^3^ c.f.u.) when the known number of serially diluted *H. pylori* was added to the infected pulp specimen, whereas the second PCR primer sets maintained a high sensitivity (Fig. 2c, d). Similar results were observed in both the first and second step PCR assays when another inflamed pulp specimen was used (data not shown). Furthermore, the sensitivities of the first and second step PCR primer sets were approximately 1–10 c.f.u. per reaction when the known number of serially diluted *H. pylori* was added to non-bacterial infected pulp specimens, though the bands in the first step PCR were weaker than those obtained from *H. pylori* genomic DNA (Fig. 2e, f). Similar results were observed in both the first and second step PCR assays when another non-infected pulp specimen was used (data not shown).

### *H. pylori* Detection in inflamed pulp specimens

**Fig. 3:**
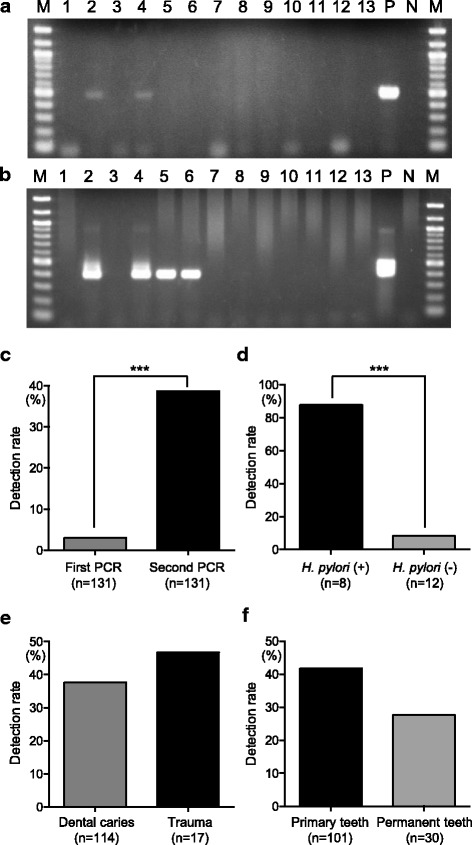
Detection of *H. pylori* from inflamed pulp specimens using single and nested PCR assays. Representative results using inflamed pulp specimens with (**a**) single and (**b**) second step PCR. Lanes 1 through 13 are specimens collected from 13 different individuals; P, *H. pylori* strains 26,695 (positive control); N, sterile water. M, molecular size marker (100 bp DNA ladder). **c** Comparison of the detection rates of *H. pylori* from inflamed pulp specimens using single and second step PCR. **d** Detection rates of *H. pylori* in the second sampling from root canal specimens. The groups were categorized as *H. pylori*-positive or -negative in the first sampling. Comparison of the detection rates of *H. pylori* from specimens obtained from dental caries verses those from trauma (**e**) or in comparison between specimens obtained from primary teeth versus permanent teeth (**f**). Significant differences were determined using Bonferroni’s method after ANOVA (***P < 0.001)

Figure 3a and b show representative results from the analyses of inflamed pulp specimens using the first and second PCR primer sets. *H. pylori* was detected in 4 of 131 (3.1%) root canal specimens in first step PCR, indicating that these samples contained more than 10^2^–10^3^ c.f.u. *H. pylori*. In second step PCR, 51 (38.9%) root canal specimens were *H. pylori*-positive, indicating that these samples contained more than 1–10 c.f.u. *H. pylori*. The nucleotide alignment of the fragment revealed that the second step PCR method could amplify the target sequence of *H. pylori*. The detection rate of *H. pylori* in second step PCR was significantly higher than that in first step PCR (***P < 0.001) (Fig. 3c). Additionally, there were no subjects in whom *H. pylori* was detected from all specimens taken from different teeth (Table 1). Among 20 specimens taken from the same teeth, 8 specimens were *H. pylori*-positive in the first sampling, 7 of which were *H. pylori*-positive in the second sampling (Fig. 3d). In addition, only 1 of 12 *H. pylori*-negative specimens showed positive results in the second sampling. There was no significant difference between the detection rate of *H. pylori* in specimens obtained from dental caries and that obtained from trauma (Fig. 3e). The detection rate of *H. pylori* in specimens obtained from primary teeth was higher than that obtained from permanent teeth, though there was no significant difference (Fig. 3f).

**Table 1:** Summary of *H. pylori* DNA detection of extirpated inflamed pulp specimens in the subjects who received root canal treatment several times

Subject	Dental caries extending pulp space (Age)				Detection Rate
1	ULA (2Y0M)	ULB (2Y1M)			0/2
	–	–			
2	URA (3Y9M)	URB (3Y9M)	ULA (3Y10M)		1/3
	+	–	–		
3	ULD (3Y10M)	URE (3Y11M)			0/2
	–	–			
4	LLC (4Y7M)	LRC (5Y5M)			1/2
	+	–			
5	LLE (5Y1M)	LLD (5Y3M)			0/2
	–	–			
6	URD (5Y2M)	LLE (5Y3M)			0/2
	–	–			
7	LLD (6Y0M)	LLE (7Y4M)			0/2
	–	–			
8	URD (6Y2M)	ULD (6Y4M)	URE (6Y5M)	LLE (6Y5M)	2/4
	+	+	–	–	
9	LLD (6Y11M)	LRE (7Y1M)			0/2
	–	–			
10	LRD (7Y4M)	URD (7Y5M)	LRD (7Y6M)		0/3
	–	–	–		
11	LLE (8Y1M)	URD (8Y2M)			0/2
	–	–			
12	LRE (7Y10M)	LRD (7Y10M)	LLE (7Y11M)	ULE (8Y0M)	2/4
	–	–	+	+	
13	LRD (8Y1M)	URD (10Y4M)			1/2
	+	–			
14	UL1 (9Y1M)	LL1 (10Y5M)			1/2
	–	+			
15	UR2 (11Y6M)	LR4 (11Y6M)	LR3 (11Y6M)	UL2 (11Y10M)	2/7
	+	–	–	–	
	UL5 (11Y10M)	UR5 (12Y1M)	UR4 (12Y1M)		
	–	+	–		
16	LL5 (14Y10M)	LL7 (14Y10M)			0/2
	–	–			

*URE* upper right primary second molar, *URD* upper right primary first molar, *URB* upper right primary lateral incisor, *URA* upper right primary central incisor, *ULA* upper left primary central incisor, *ULB* upper left primary lateral incisor, *ULD* upper left primary first molar, *ULE* upper left primary second molar, *LLE* lower left primary second molar, *LLD* lower left primary first molar, *LLC* lower left primary canine, *LRC* lower right primary canine, *LRD* lower right primary first molar, *LRE* lower right primary second molar, *UR5* upper left second premolar, *UR4* upper right first premolar, *UR2* upper right lateral incisor, *UL1* upper left central incisor, *UL2* upper left lateral incisor, *UL4* upper left first premolar, *UL5* l upper left second premolar, *LL7* lower left second molar, *LL5* lower left second premolar, *LL1* lower left central incisor, *LR3* lower right canine

### *H. pylori* Adhesion to HDPFs

**Fig. 4:**
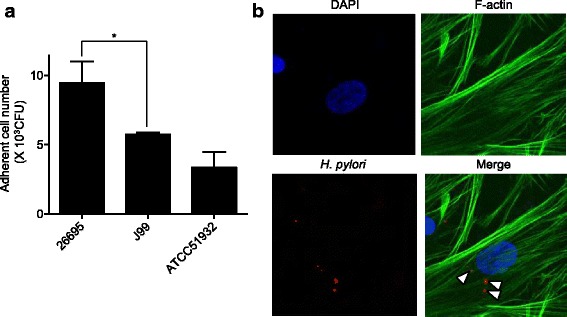
Adhesion of *H. pylori* strains to human dental pulp fibroblast cells. **a** Adhesion rates were calculated based on the ratio of recovered to infected strains with an multiplicity of infection of 10. Significant differences were determined using Bonferroni’s method after ANOVA (*P < 0.05). **b** Representative confocal scanning laser microscopic images of *H. pylori* strain 26,695 showing adhesion to human dental fibroblast cells following dual labeling. Nuclei are stained blue, bacterial cells adhering to HDPFs are stained red (arrows), and actin filaments are stained green

All three tested *H. pylori* strains showed adhesion to HDPFs (Fig. 4a). Among these strains, 26,695 showed significantly higher adhesion rates than J99 (*P < 0.05). Confocal scanning laser microscopy demonstrated that *H. pylori* strain 26,695 clearly adhered to HDPFs (Fig. 4b).

## Discussion

The reliable PCR system for the *H. pylori* detection in oral specimens requires high specificity and sensitivity levels since it is known that there are approximately 700 bacterial phylotypes found in the oral cavity [17]. To overcome difficulties in detecting *H. pylori* in the oral cavity, we previously designed novel primer sets using the complete genome information for 48 *H. pylori* strains registered in the GenBank database [5]. In that study, we found six sequences of at least 20 consecutive nucleotides conserved among all strains in the *ureA* gene. The *ureA* gene is known to encode the urease enzyme, which has been frequently used to develop PCR primers for *H. pylori* detection [7, 10, 18]. However, no previous *ureA*-based primers were completely conserved among all strains registered in the database [5].

We previously constructed five primer sets (*ureA*-aF/aR, *ureA*-aF/bR, *ureA*-bF/aR, *ureA*-bF/bR, *ureA*-cF/cR) for the detection of *H. pylori* based on six sequences of at least 20 consecutive nucleotides [5]. These primer sets showed an appropriate level of sensitivity at approximately 1–10 c.f.u. per reaction for *H. pylori* genomic DNA. However, these primers sets revealed a sensitivity of approximately 10^2^–10^3^ cells for the detection of *H. pylori* DNA from inflamed pulp specimens. It has been reported that some PCR primers sets showed lower detection limits in *H. pylori* from gastric tissues or oral specimens compared with that from *H. pylori* genomic DNA [11]. In addition, we compared the sensitivities of detecting *H. pylori* from infected pulp specimens versus non-infected pulp specimens by adding a known number of serially diluted *H. pylori* to these specimens using the single PCR methods. The sensitivity in inflamed pulp specimens was much lower than that in non-infected pulp specimens, which indicated that numerous bacteria present at more than 10^6^ c.f.u. in inflamed pulp may be the main cause for difficulty in detecting *H. pylori* in such specimens. Thus, we refined our system using a nested PCR method to improve the sensitivity for detection of *H. pylori* DNA from inflamed pulp tissue. Among the five primer sets beased on the *ureA* gene designed in our previous study, primer sets *ureA*-aF/bR and *ureA*-bF/aR were suitable for the nested PCR method.

Nested PCR is one of the most sensitive methods to detect a small number of bacteria from clinical specimens [19]. The specificity and sensitivity of target DNA amplification is estimated to be approximately 1000–10,000 times more sensitive than standard PCR [20]. Some researchers previously reported *H. pylori* detection using nested PCR methods [21]. However, complete genome information was unavailable when these nested PCR methods were reported. *H. pylori* 26,695 (ATCC 700392) is the first strain reported to identify the complete genome sequence in 1997, then only several complete genome sequences of *H. pylori* strains were reported until 10 years ago [22]. To our knowledge, this is the first study to propose a nested PCR method designed based on a large amount of genome information.

The detection rate of *H. pylori* from inflamed pulp specimens detected by single PCR with primer sets *ureA*-aF/*ureA*-bR were 3.0%, whereas that with primer set *ureA*-aF/*ureA*-aR was 15% [5]. However, the detection rate using primer set *ureA*-aF/*ureA*-aR was based on the results from only 40 specimens. Thus, the detection rate of the primer set *ureA*-aF/*ureA*-aR in 131 specimens was investigated in the present study, resulting in a detection rate of 5.3% (data not shown). Though the detection rate of *H. pylori* with primer set *ureA*-aF/*ureA*-aR was higher than that with primer set *ureA*-aF/*ureA*-bR, which may be because the primer set *ureA*-aF/*ureA*-aR was the most sensitive of all primer combinations [5], the detection limits of both primer sets were similar (approximately 1–10 cells using *H. pylori* genomic DNA). The single PCR method using primer set *ureA*-aF/*ureA*-aR is a simple and highly sensitive method; however, nested PCR is the most appropriate method for *H. pylori* detection because it showed highest sensitivity (1–10 cells) and detection rate (38.9%) in inflamed pulp specimens.

To determine the detailed distribution of *H. pylori* in inflamed pulp tissue, we analyzed the detection rate of *H. pylori* in 16 subjects who received root canal treatment in different teeth. In addition, *H. pylori* detection in 20 subjects with two samplings from the same tooth was performed. *H. pylori* was not detected from all inflamed pulp specimens in each individual, indicating that *H. pylori* was not widely distributed in teeth with infected root canals. However, most *H. pylori*-positive specimens were positive in the second sampling, suggesting that *H. pylori* was not transiently present but rather colonized the inflamed pulp tissue. Since the intervals between first and second samplings were approximately 1–2 weeks in most subjects, the precise colonization period of *H. pylori* in infected root canal remains unknown. Further studies should be performed to clarify this point. Comparison of the detection rates of *H. pylori* from specimens obtained from primary teeth versus permanent teeth revealed that higher detection rate of *H. pylori* was observed in specimens obtained from primary teeth. The result may be reasonable because *H. pylori* infections seem to be acquired in childhood [2].

Since *H. pylori* was not transiently present in infected root canal, we investigated the mechanism by which *H. pylori* colonizes pulp tissue by analyzing the adhesion property of *H. pylori* to HDPFs. Bacterial adhesion to host cells is considered an important virulence factor for many bacterial species [23]. For *H. pylori*, bacterial adhesion to gastric epithelium is a critical factor for *H. pylori* colonization [24]. However, there have been few reports focusing on *H. pylori* adhesion to cells obtained from oral origin, and, to our knowledge, this is the first study to investigate the adhesion properties of *H. pylori* to HDPFs. All three tested *H. pylori* strains showed adhesion to HDPFs, which is likely one of the reasons why *H. pylori* DNA was detected in inflamed pulp specimens. Among these strains, *cagA*-positive *H. pylori* strains 26,695 and J99 showed higher adhesion rates compared with *cagA*-negative *H. pylori* strain ATCC 51932. The *cagA* gene belongs to the *cag* pathogenicity island (PAI) [25]. The *cag* PAI contains several virulence genes, some of which were reported to be related to *H. pylori* adhesion to gastric epithelial cells [26, 27]. Though *cagA* is one of the possible genes related to *H. pylori* adhesion, various *H. pylori* adhesins have also been reported [28], and the adhesion mechanisms to HDPFs remain to be elucidated. Further studies should be performed to investigate bacterial adhesion induced by *H. pylori* virulence genes.

## Conclusions

In summary, our nested PCR approach to detect *H. pylori* in inflamed pulp was constructed using two primers sets, both of which were designed based on conserved sequences in *H. pylori* strains. The nested PCR method showed a higher sensitivity than the single PCR method, which used only one primer set. In addition, *H. pylori* strains showed adhesion to HDPFs. These results clearly suggest that *H. pylori* may colonize dental pulp tissue.

## Abbreviations

HDPFs: Human dental fibroblast cells
PBS: Phosphate-buffered saline
